# Edible mushrooms as emerging biofactories for natural therapeutics and oral biopharmaceutical delivery

**DOI:** 10.3389/ffunb.2025.1742455

**Published:** 2025-12-18

**Authors:** Kevin Wang, Annie Wang, Byron Meade

**Affiliations:** Division of Math and Natural Sciences, University of Pikeville, Pikeville, KY, United States

**Keywords:** edible fungi, fungal biotechnology, mushroom molecular farming, oral biopharmaceutical delivery, recombinant protein expression

## Abstract

Mushrooms have long served as both food and medicine, providing polysaccharides, terpenoids, phenolics, and peptides with diverse health benefits. Extensive studies have begun to clarify the molecular mechanisms underlying these therapeutic effects, which include anti-aging, immunomodulatory, anticancer, and anti-inflammatory activities. Translational research is progressing from preclinical models to clinical trials, reinforcing the biomedical potential of mushroom-derived compounds. Advances in fungal genetic modification and gene editing have further positioned edible mushrooms as promising platforms for recombinant biopharmaceutical production. Their eukaryotic protein-processing capacity, natural bioencapsulation, and GRAS (Generally Recognized as Safe) status make them well-suited for sustainable and orally deliverable therapeutics. Engineered mushrooms show strong potential as platforms for oral vaccines and recombinant protein production, bridging traditional medicinal use with modern molecular farming.

## 1 Introduction

Mushrooms are increasingly recognized for their exceptional nutritional and medicinal potential. They provide essential amino acids, vitamins, minerals, and a wide array of bioactive molecules that modulate immune, metabolic, and neurological functions (Barua et al., 2024; Bell et al., 2025). Modern biomedical research has renewed interest in mushrooms as prolific sources of pharmacologically active compounds with antitumor, antioxidant, immunomodulatory, and antiviral properties (Njue et al., 2024; Doonan et al., 2025).

Preclinical studies have demonstrated that β-glucans such as lentinan from *Lentinula edodes* and D-fraction from *Grifola frondosa*, triterpenoids from *Ganoderma lucidum*, and cordycepin from *Cordyceps militaris* suppress tumor growth, enhance immune surveillance, and sensitize cancer cells to chemotherapy (Lin et al., 2024; Thepmalee et al., 2024; Zhou et al., 2024). Clinically, polysaccharide preparations including lentinan and the protein-bound polysaccharides PSK and PSP from *Trametes* (*Coriolus*) *versicolor* have been used as chemotherapy adjuvants. Meta-analyses and randomized clinical trials report improved immune parameters and, in some cases, prolonged progression-free or overall survival (Sivanesan et al., 2022; Jędrzejewski et al., 2023; Zhou et al., 2024).

Although molecular farming research using mushrooms is still in its early stages, recent advances in fungal transformation systems (Liu et al., 2019, Liu et al., 2020; Cai et al., 2024; Yin and Xiong, 2025) and CRISPR/Cas-based genome editing and synthetic tool kits (Koshi et al., 2022; Liu et al., 2023; Koshi et al., 2024; Maini et al., 2024) have opened new opportunities for developing mushrooms as biopharmaceutical hosts. These innovations suggest that mushrooms are progressing from traditional medicinal resources to next-generation biofactories for the sustainable production of recombinant proteins and therapeutic metabolites, marking a new phase in fungal molecular farming and translational biotechnology.

## 2 Therapeutic potential of mushrooms: disease-oriented perspectives

**Figure 1:**
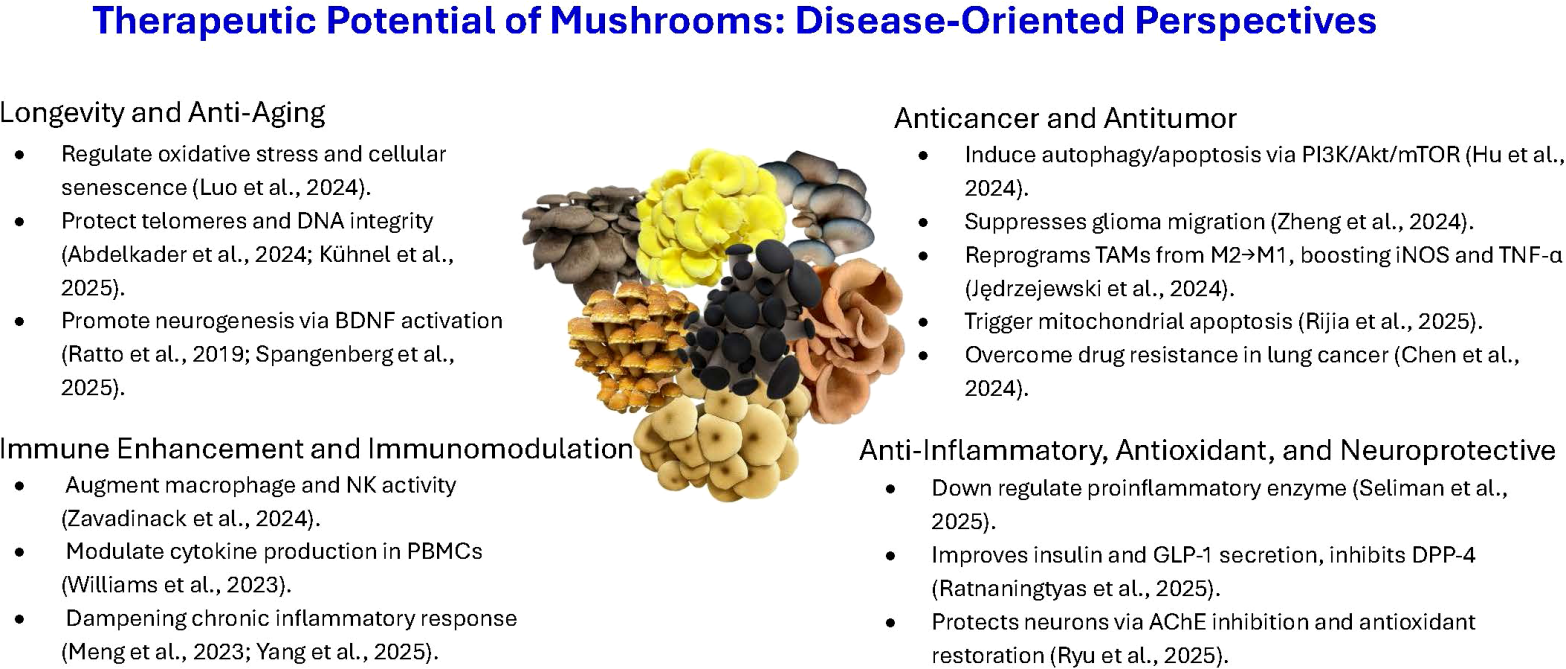
Therapeutic potential of mushrooms: disease-oriented perspectives. This infographic highlights the principal therapeutic functions of medicinal and edible mushrooms and their underlying molecular mechanisms, encompassing anti-aging, immunomodulatory, anticancer, anti-inflammatory, antioxidant, and neuroprotective effects.

The medicinal properties of mushrooms were traditionally recognized through empirical use, with limited understanding of their molecular basis. Recent research has begun to clarify these mechanisms, revealing that key bioactive compounds such as polysaccharides, terpenoids, phenolics, and proteins or peptides can modulate immune, inflammatory, and metabolic pathways. These discoveries have transformed mushrooms from traditional health remedies into important subjects of modern biochemical and pharmacological research, supporting their development as therapeutic and nutraceutical agents (**Figure 1**).

### 2.1 Longevity and anti-aging effects

Mushrooms such as *G. lucidum* (Reishi) and *C. sinensis/militaris* have long been regarded as natural “elixirs of life.” Modern research increasingly supports these traditional perspectives, showing that mushroom-derived metabolites help regulate oxidative stress, enhance mitochondrial function, and delay cellular senescence, thereby promoting healthy aging (Luo et al., 2024).

Mechanistic studies have revealed these anti-aging effects across multiple species. Extracts from *Agaricus bisporus*, *Pleurotus ostreatus*, *G. lucidum*, and *Hericium erinaceus* improve fibroblast viability under oxidative stress, decrease reactive oxygen species (ROS), and preserve telomere length (Abdelkader et al., 2024). Psilocin, the active metabolite of psilocybin, activates the SIRT1 and Nrf2 pathways while suppressing senescence-associated markers, thereby preserving mitochondrial DNA integrity and extending lifespan in aged mice (Kato et al., 2025). Likewise, ergothioneine (EGT) from *L. edodes* protects neurons against oxidative damage by stimulating Nrf2 signaling and maintaining mitochondrial stability (Apparoo et al., 2024).

Further studies demonstrate that *G. lucidum* polysaccharides and triterpenoids mitigate cardiac aging by activating the Nrf2/HO-1 and SOD2 antioxidant pathways, thereby enhancing cellular defense and mitochondrial stability (Kühnel et al., 2025). Likewise, extracts of *H. erinaceus* enriched in erinacines and hericenones stimulate neurogenesis and improve cognitive performance by activating brain-derived neurotrophic factor (BDNF) and Nrf2 signaling, while concurrently reducing inflammation and amyloid-β accumulation (Ratto et al., 2019; Spangenberg et al., 2025). These findings suggest that mushroom-derived metabolites restore redox balance, sustain telomerase activity, and strengthen mitochondrial and neural resilience, highlighting their potential as natural modulators of longevity and cognitive health.

### 2.2 Immune enhancement and immunomodulation

Mushrooms function as powerful biological response modifiers through their complex repertoire of polysaccharides, lectins, and proteins that influence both innate and adaptive immunity. These bioactives activate macrophages, dendritic cells, and natural killer (NK) cells, thereby strengthening immune surveillance. For example, β-glucan- and galactan-rich polysaccharides from *Lactarius quieticolor* enhanced nitric-oxide production and macrophage differentiation (Zavadinack et al., 2024), while a mannose-binding lectin from *A. bisporus* promoted phagocytosis and cytokine secretion without cytotoxic effects (Ismaya et al., 2024).

Mushroom extracts also modulate inflammatory and antiviral signaling networks. Preparations from *Hericium coralloides* and *T. versicolor* rebalanced cytokine profiles in immune cells from older adults, reducing IL-5 and IL-13 while adjusting IL-1β, IL-6, and interferon levels—an effect relevant to age-related immune dysfunction (Williams et al., 2023). Likewise, *G. lucidum* polysaccharide peptides attenuated rheumatoid arthritis by inhibiting NF-κB and MAPK pathways, lowering pro-inflammatory cytokines, and restoring immune equilibrium (Meng et al., 2023).

Proteomic analyses have shown that many edible mushrooms produce immunoregulatory proteins that enhance macrophage activity, stimulate cytokine release, and promote M1 polarization (Xu et al., 2023). In autoimmune models, cordycepin from *Cordyceps* species reduced interferon-driven inflammation by promoting STING degradation through autophagy (Yang et al., 2025). Overall, mushroom-derived biomolecules orchestrate complex immune regulation by balancing activation and suppression pathways and show strong potential as food-based immunonutraceuticals and therapeutic adjuncts for inflammatory and autoimmune diseases.

### 2.3 Anticancer and antitumor activities

Mushrooms are a rich source of structurally diverse metabolites with strong anticancer and antitumor potential. Bioactive compounds, such as polysaccharides, triterpenoids, sterols, and phenolics, exert effects through multiple mechanisms such as apoptosis induction, autophagy modulation, immune activation, and inhibition of angiogenesis and metastasis.

Among these metabolites, polysaccharides have been the most extensively characterized. A rhamnogalactan (HTP) from *L. quieticolor* exhibited cytotoxicity against triple-negative breast cancer (TNBC) cells and synergized with paclitaxel, suggesting value as a chemotherapeutic adjuvant (de Lara et al., 2025). β-D-glucan (LNT) from *L. edodes* induced Ca²^+^-dependent autophagy through the PI3K/Akt/mTOR pathway while promoting apoptosis in cervical cancer cells, and autophagy inhibition further enhanced its cytotoxicity (Hu et al., 2024). A heteropolysaccharide (SCFP) from *Schizophyllum commune* suppressed glioma growth by upregulating ARHI and inhibiting PI3K/Akt signaling, demonstrating polysaccharide-mediated reprogramming of tumor metabolism (Zheng et al., 2024).

Other mushroom-derived compounds, including triterpenoids and sterols, also show potent anticancer properties. Methanolic extracts of *Ganoderma applanatum* triggered mitochondrial dysfunction and DNA fragmentation in hepatocellular carcinoma cells, leading to apoptosis with minimal toxicity (Rijia et al., 2025). Ergostane-type steroids from *T. versicolor* exhibited strong affinity for key oncogenic targets such as CDK2, Bcl-2, and VEGFR-2 (Njue et al., 2024).

Immunomodulatory effects further contribute to their therapeutic potential: *C. versicolor* extracts reprogrammed tumor-associated macrophages from M2 to M1 phenotypes, restoring antitumor immune surveillance in TNBC models (Jędrzejewski et al., 2024), while the triterpenoid GA-DM from *G. lucidum* activated apoptosis and autophagy in B-cell lymphoma with low systemic toxicity (Doonan et al., 2025). Advances in nanobiotechnology have also enhanced the efficacy of mushroom metabolites; for example, selenium nanoparticles stabilized by *Agrocybe aegerita* protein overcame drug resistance in lung cancer, and glucomannan from *S. commune* inhibited the PI3K/Akt/mTOR pathway with high structural stability (Chen et al., 2024; Gao et al., 2024). These studies together underscore the promise of mushrooms as sources of multifunctional, low-toxicity anticancer agents and adjuvants for modern cancer therapy.

### 2.4 Other therapeutic applications: anti-inflammatory, antioxidant, and neuroprotective effects

Chronic inflammation and oxidative stress are key contributors to many degenerative and metabolic disorders. Mushroom-derived compounds help restore physiological balance by modulating cytokine signaling, neutralizing reactive oxygen species (ROS), and activating endogenous antioxidant defenses. *G. lucidum* extract also restored redox balance under hypoxic stress by normalizing energy ratios (NAD/NADH, ADP/ATP) and modulating NF-κB, IL-6, and TNF-α expression while enhancing Nrf2 and HIF-1α signaling (Maithani and Tulsawani, 2025). Additionally, a lectin (AVL) from *Amanita vaginata* downregulated pro-inflammatory enzymes, suggesting potential as a novel lectin-based anti-inflammatory agent (Seliman et al., 2025).

Mushrooms are exceptional sources of natural antioxidants and metabolic regulators. *Boletus edulis* was identified as a rich source of selenoneine, a selenium analogue of ergothioneine, providing highly bioavailable antioxidant selenium (Peer and Kuehnelt, 2024). Preservation methods such as microwave-vacuum drying maintain high levels of phenolic and ergothioneine content in *Cyttaria espinosae*, thereby enhancing its antioxidant capacity (Villalobos-Pezos et al., 2024). In diabetic models, microencapsulated *Pleurotus cystidiosus* improved glucose metabolism and reduced inflammation by lowering TNF-α and IL-1β, increasing insulin and GLP-1 secretion, and inhibiting DPP-4 activity, demonstrating combined antidiabetic and anti-inflammatory benefits (Ratnaningtyas et al., 2025).

Several mushrooms also exhibit potent neuroprotective and cognitive-enhancing properties. *Pleurotus citrinopileatus* extract protected neurons from oxidative apoptosis and improved memory in mice by inhibiting acetylcholinesterase and boosting antioxidant enzymes (Ryu et al., 2025). Combined extracts of *G. lucidum*, *H. erinaceus*, *P. ostreatus*, and *A. bisporus* reduced oxidative stress, preserved telomeres, and balanced cytokine profiles in aging models (Abdelkader et al., 2024; Abdelkader et al., 2025). Moreover, fungal meroterpenoids, hybrid polyketide-terpenoid metabolites, exhibit anti-cholinesterase and BACE1-inhibitory activity, offering promising leads for Alzheimer’s and Parkinson’s disease therapy (Dimitrova et al., 2025). Overall, mushroom metabolites function as multifunctional regulators of inflammation, oxidation, and neurodegeneration, supporting their development as safe, food-based interventions for chronic metabolic and neurological disorders.

### 2.5 From preclinical promise to clinical translation

Extensive preclinical studies have demonstrated that mushrooms are rich sources of pharmacologically active biomolecules, including β-glucans, triterpenoids, polysaccharide–protein complexes, lectins, and ergothioneine. These compounds regulate immune activity, inhibit tumor growth, restore oxidative balance, and improve metabolic homeostasis through multiple molecular pathways. Despite this solid experimental foundation, clinical evaluation remains limited due to variations in extraction procedures, compound complexity, dosage inconsistencies, and the lack of standardized formulations.

Nevertheless, mushroom-derived therapeutics are gaining recognition in evidence-based medicine. An increasing number of formulations have advanced to Phase I–III clinical trials exploring their roles in immune enhancement, inflammation control, cancer adjuvant therapy, and antiviral protection. **Table 1** presents Phase I-III human clinical studies of medicinal and edible mushroom preparations. Preclinical and observational research were excluded to maintain a focus on trials that directly evaluate clinical safety and efficacy.

**Table 1:** Representative clinical trials involving medicinal and edible mushrooms.

Trial ID	Mushroom/Extract	Condition/Population	Design/Phase	Primary endpoints
NCT02814617	*C. sinensis* mycelium (CBG-CS-2)	Healthy adults	Phase II/III	Immune enhancement via NK and cytokine response
NCT06028022	*G. lucidum* (Reishi)	Breast cancer patients on aromatase inhibitors	Phase II/Recruiting	Reduced fatigue and inflammation
NCT00709020	*A. bisporus* (White Button Mushroom)	Breast-cancer survivors	Phase I/Completed	Safety and recurrence prevention
NCT04519879	*A. bisporus* supplement	Prostate cancer patients	Phase II/Active, not recruiting	Reduced PSA, immune modulation
NCT01496053	*Agaricus blazei* (AndoSan™ extract)	Ulcerative colitis patients	Phase II/III, Completed	Reduced inflammation and improved quality of life
NCT00680667	*T. versicolor* (Turkey Tail)	Women with breast cancer post-radiotherapy	Phase I/Completed	Improved immune function, reduced fatigue
NCT06450873	*T. versicolor* extract	Postmenopausal ER+ breast cancer	Phase II/Ongoing	Immune modulation and safety
NCT02405533	AHCC^®^ (*L. edodes* mycelia extract)	Women with persistent HPV infection	Phase II/Completed	HPV clearance and immune enhancement
NCT05763199	AHCC^®^ + Chemotherapy	Ovarian cancer patients	Phas III/Recruiting	Reduced chemo toxicity, improved QOL
NCT00687843	*T. versicolor* PSK + TS-1	Gastric cancer	Phase III/Completed	Improved survival and immune parameters
NCT00069524	*P. ostreatus* (Oyster mushroom)	HIV patients with hyperlipidemia	Phase I/II Completed	Improved lipid profile, antioxidant effect
NCT04210336	*C. versicolor* (Papilocare^®^ vaginal gel)	HPV-related cervical lesions	Phase III/Completed	Regression of HPV-induced lesions

### 2.6 Mechanistic overview box

Mushroom-derived bioactive compounds exert therapeutic effects through several conserved molecular pathways that regulate inflammation, oxidative stress, immune activation, neuroprotection, and cancer progression. Key mechanisms include activation of the Nrf2/HO-1/SOD2 antioxidant pathway; inhibition of NF-κB and MAPK signaling; modulation of STING-mediated interferon responses; regulation of PI3K/AKT/mTOR signaling influencing apoptosis and autophagy; macrophage activation and polarization; and enhancement of BDNF-related neurogenic signaling. These shared pathways provide a unified mechanistic framework that supports the diverse therapeutic activities of medicinal and edible mushrooms.

Despite growing interest in mushroom-derived therapeutics, existing clinical studies remain limited by several important constraints. Many human trials are underpowered because early-phase studies typically enroll only 20–80 participants, as demonstrated by trials of *G. lucidum* for cancer-related fatigue (NCT06028022) and *A. bisporus* extracts in breast cancer survivors (NCT00709020). Such small cohorts reduce statistical power and restrict the generalizability of clinical findings. Extraction methods, strain sources, and formulation strategies also vary widely across studies. For example, polysaccharide–protein complexes such as PSK and PSP from *T. versicolor* differ substantially in preparation methods, biochemical composition, and dosing across trials (Sivanesan et al., 2022; Jędrzejewski et al., 2023), while AHCC^®^ products derived from *L. edodes* mycelia exhibit batch-to-batch variability that complicates cross-study comparisons (NCT02405533). Differences in preparation formats—including hot-water extracts, fermented mycelial biomass, ethanol-derived triterpenoids, and purified β-glucans—further challenge the standardization of dose, purity, and bioactive content.

In addition, most clinical studies involve short treatment durations (often 4–12 weeks) or limited follow-up, as seen in trials evaluating *C. sinensis* mycelial powder (NCT02814617) or *P. ostreatus* for metabolic regulation (NCT00069524). These design features restrict assessments of long-term safety and sustained efficacy. Variability in compound characterization further limits comparability across studies. For example, lentinan preparations differ widely in molecular weight, β-glucan branching structure, purity, and extraction methods, all of which influence immunomodulatory activity (Zhou et al., 2024). Collectively, these differences in chemical composition, analytical reporting, and clinical endpoints make it difficult to harmonize data across trials or perform rigorous meta-analyses.

## 3 From wild medicine to molecular farming

The convergence of traditional mycotherapy and modern biotechnology has opened a new frontier in mushroom molecular farming. Traditionally, molecular farming referred to the use of plants as biofactories for producing recombinant proteins, enzymes, and vaccines (Akher et al., 2025). Mushrooms offer comparable scalability and safety advantages while providing unique benefits for the development of orally deliverable biopharmaceuticals. Edible and medicinal species have recently emerged as promising extensions of this concept, combining advanced eukaryotic protein-folding and post-translational modification capabilities with GRAS status. These features make mushrooms highly attractive for food-grade biopharmaceutical production and oral therapeutic delivery.

### 3.1 Advantages of mushroom molecular farming

Mushrooms offer multiple intrinsic advantages as biofactories for recombinant oral therapeutics. Their multicellular structure, comprising mycelia, fruiting bodies, and spores, enables spatial and temporal control of transgene expression. Unlike unicellular yeasts, which have limited capacity for complex and mammalian-like post-translational processing, mushrooms can perform advanced modifications such as glycosylation, phosphorylation, and disulfide-bond formation, resulting in correctly folded and biologically active proteins. Their edible nature and cultural acceptance also reduce downstream purification requirements, making direct oral administration of bioencapsulated recombinant products feasible.

Proof-of-concept studies have validated this potential. *Coprinopsis cinerea* successfully expressed a fungal immunomodulatory protein (FIP-gsi) from *Ganoderma sinense* at approximately 0.3 mg g^-^¹ fresh mycelium while retaining full bioactivity (Han et al., 2010). *Flammulina velutipes* was engineered to produce enterovirus 71 virus-like particles (EV71-VLPs) (Lin et al., 2015), and later, hepatitis B surface antigen (HBsAg) expressed in its fruiting bodies elicited protective immune responses in pigs (Huang et al., 2019). These studies confirm that mushrooms are safe, scalable, and cost-effective platforms for the production of recombinant vaccines and therapeutic proteins.

### 3.2 Edible bioreactors and oral delivery

Edible mushrooms possess distinctive biological and structural features that make them promising bioreactors for recombinant protein production and oral delivery. Their fruiting bodies provide abundant, protein-rich biomass that can be directly consumed or processed into encapsulated formulations. Mushroom cells naturally bioencapsulate recombinant proteins within their cellular matrix, protecting them from gastric degradation and enabling mucosal immune stimulation following oral administration. In addition, intrinsic β-glucans and polysaccharide–protein complexes function as natural adjuvants, amplifying immune responses against co-expressed antigens (Lin et al., 2015; Huang et al., 2019; Zou et al., 2024).

Species such as *G. lucidum*, *L. edodes*, *P. ostreatus*, and *Tremella fuciformis* combine strong immunostimulatory properties with genetic tractability, supporting the design of dual-function edible vaccines that deliver both antigen and adjuvant within a single organism (Rahman and Rahman, 2024). Such mushroom-based vaccine platforms could greatly enhance accessibility by eliminating the need for cold-chain storage and injections, providing shelf-stable, nutritionally beneficial formulations suitable for distribution in low-resource regions.

**Figure 2:**
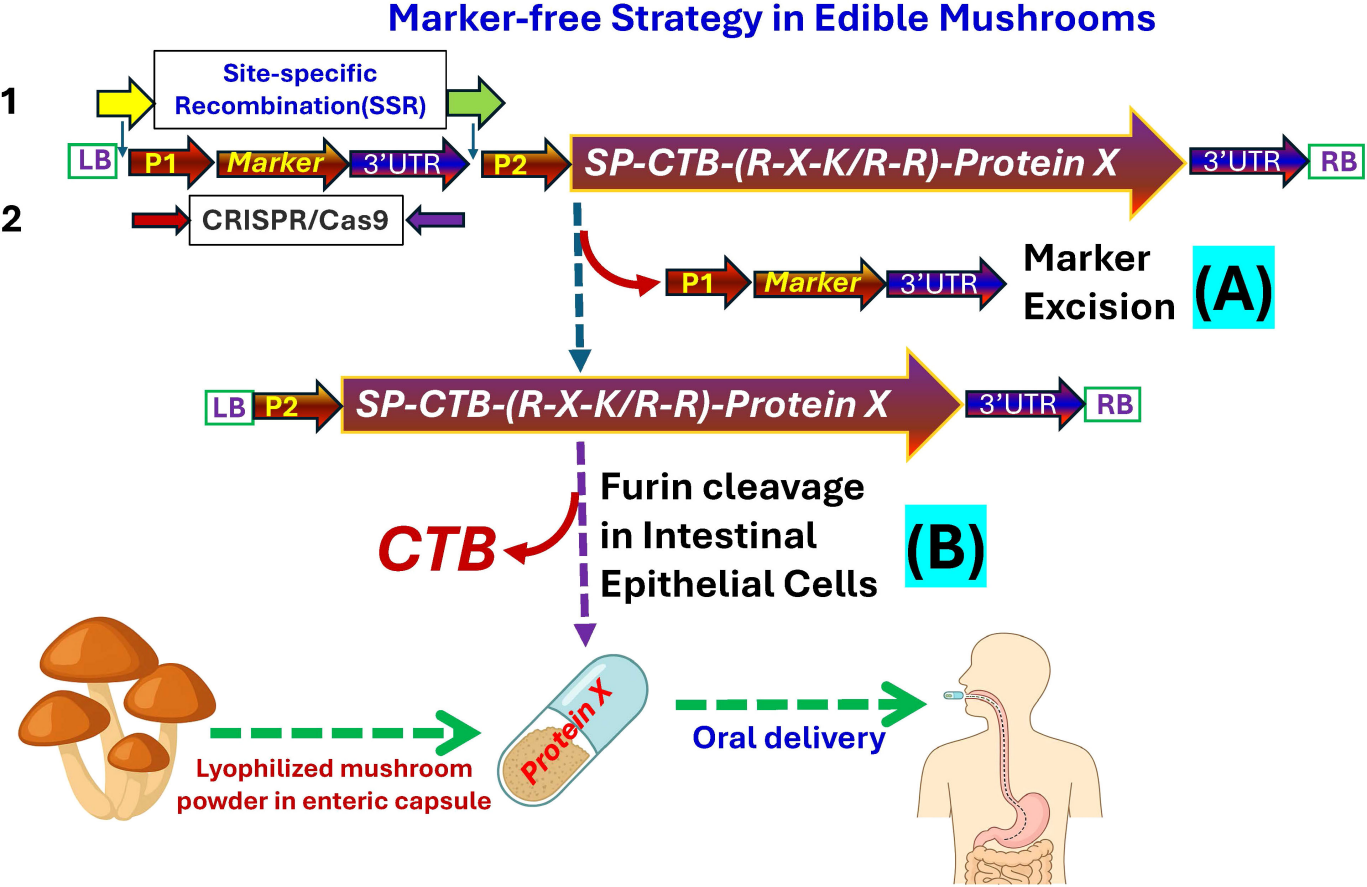
Marker-free molecular farming strategy in edible mushrooms. Schematic representation of a marker-free transformation system for edible mushrooms. The construct contains promoter P1 driving a selectable marker and promoter P2 controlling a fusion gene consisting of a signal peptide (SP), CTB, a furin-cleavage motif (R-X-K/R-R), and the therapeutic protein (X). The marker gene is flanked by site-specific recombination (SSR) recognition sites for precise excision (**1**) or can be removed using CRISPR/Cas9 editing (**2**). After transformation and selection, transient recombinase expression eliminates the marker, generating a clean, food-grade recombinant line **(A)**. The engineered mushrooms produce bioencapsulated therapeutic proteins that are lyophilized and formulated into enteric-coated capsules for oral delivery. Following ingestion, CTB mediates GM1 receptor binding and intestinal uptake, and furin cleavage activates the therapeutic protein systemically **(B)**. LB and RB indicate the left and right T-DNA borders of the *Agrobacterium* Ti plasmid.

Although early studies have focused on vaccines, the same strategies can be applied to the production of therapeutic proteins. Edible mushrooms share key eukaryotic protein-processing and post-translational capabilities with higher plants (Lübeck and Lübeck, 2022) and can adopt bioencapsulation strategies pioneered in lettuce systems (Ding et al., 2025). Engineering recombinant proteins with signal peptides, furin-cleavage motifs (R-X-K/R-R), and transmucosal carriers such as the cholera toxin B subunit (CTB) can facilitate epithelial transcytosis and systemic delivery. The CTB domain mediates GM1 receptor binding, while the furin motif enables proteolytic activation following uptake. This approach offers a pathway for developing freeze-dried, food-grade mushroom powders that can be encapsulated in enteric-coated capsules for safe, efficient, and orally deliverable biopharmaceuticals (**Figure 2**).

### 3.3 Marker-free genetic engineering of edible mushrooms for oral biopharmaceutical production

The next step in advancing mushroom molecular farming is the development of marker-free, food-grade transformation systems tailored for oral biopharmaceutical delivery. Conventional fungal transformation relies on antibiotic or herbicide-resistance genes (e.g., *hph*, and *nptII*) for selection. Although effective experimentally, such markers raise biosafety and regulatory concerns for edible applications. To ensure consumer safety and GRAS compliance, the final recombinant strains must be marker-free, without any foreign resistance genes or plasmid sequences (Koshi et al., 2022, Koshi et al., 2024).

Modern strategies for marker elimination include self-excising selection systems, inducible site-specific recombination, and DNA-free genome editing. The trans-nuclei CRISPR/Cas9 system enables transient, DNA-free genome editing, producing clean, “footprint-free” mushroom transformants suitable for food-grade and GRAS-compliant applications (Koshi et al., 2022, Koshi et al., 2024). Meanwhile, recombinase-based systems such as Cre/loxP or phiC31 integrase, widely applied in transgenic plant, enable precise removal of selectable markers following stable transgene integration (Wang et al., 2011). When combined with inducible promoters responsive to heat, chemical inducers, or transient expression of recombinase, these systems allow controlled marker excision, leaving only the target expression cassette (**Figure 2**).

For oral delivery, marker-free mushrooms can be engineered to express therapeutic proteins fused to transmucosal carriers, such as CTB, and to include furin-cleavage motifs for post-absorption activation. Once produced in mycelium or fruiting tissue, recombinant proteins can be freeze-dried and encapsulated into enteric-coated capsules, protecting the payload through gastric transit and releasing it in the intestine, where uptake occurs via the mucosal surface.

This clean-label molecular farming framework provides both regulatory and ethical advantages, aligning with global trends in precision food-safe biotechnology. As innovations in CRISPR/Cas, excision technologies, and metabolic pathway optimization advance, edible mushrooms could become self-contained biofactories that express, encapsulate, and deliver therapeutic proteins directly through oral routes—offering a sustainable, low-cost alternative to conventional injectable biologics.

### 3.4 Challenges and limitations

Despite significant progress, mushroom molecular farming continues to face technical and biological challenges. Transformation efficiency remains low due to the rigid chitin-rich cell wall, heterogeneous tissue organization, and complex life cycles, which hinder DNA delivery and stable integration. Recombinant protein yields are further constrained by weak promoters, transcriptional silencing, and proteolytic degradation, while inconsistent glycosylation and post-translational variability can compromise protein stability and bioactivity. Recent advances, including CRISPR/Cas-based genome editing (Koshi et al., 2022, Koshi et al., 2024), redesign of regulatory elements (Zhang et al., 2022), and optimization of post-translational factors (Guo et al., 2025), are addressing these limitations. Moreover, the introduction of modular synthetic-biology toolkits for edible fungi (Maini et al., 2024) enables rapid construct assembly and improved control over recombinant expression, paving the way for reliable, scalable, and food-grade mushroom platforms for oral biopharmaceutical production.

### 3.5 Perspectives

Mushroom molecular farming represents a transformative synthesis of traditional mycotherapy and synthetic biology, providing an ecologically sustainable platform for oral biopharmaceuticals. Their combination of precise protein processing, natural bioencapsulation, and low production cost aligns with global priorities for accessible, cold-chain–independent therapeutics. The future integration of automated cultivation, lyophilization, and a marker-free strategy will accelerate the realization of edible fungal bioreactors capable of producing vaccines, enzymes, and therapeutic recombinant proteins for oral administration. By bridging agriculture, biotechnology, and medicine, edible mushrooms stand at the forefront of next-generation molecular farming, offering safe, affordable, and nutritionally integrated solutions for global healthcare.

## 4 Conclusions and future outlook

Mushrooms have evolved from ancient natural remedies into modern platforms for biopharmaceutical innovation. Their rich portfolio of bioactive metabolites supports therapeutic use across cancer, aging, and neurodegenerative disorders, while their genetic and metabolic versatility positions them as sustainable molecular-farming hosts. Edible mushrooms, with intrinsic GRAS status and natural encapsulation capacity, provide an unprecedented opportunity for oral delivery of recombinant proteins and vaccines. Achieving this vision will require interdisciplinary collaboration, standardized transformation protocols, and clear regulatory frameworks for edible genetically engineered fungi. Continued advances in fungal biotechnology and oral delivery design will cement mushrooms as key players in the future of accessible, sustainable, and nutritionally integrated biopharmaceuticals.
